# Evaluation of the effects of fear and anxiety on nutrition during the COVID-19 pandemic in Turkey

**DOI:** 10.1017/S1368980020003845

**Published:** 2020-09-25

**Authors:** Seda Kaya, Zeynep Uzdil, Funda Pinar Cakiroğlu

**Affiliations:** 1 Faculty of Health Sciences, Department of Nutrition and Dietetics, Ankara University, Tepebaşi Neighborhood, Fatih Street, No: 197 Keçiören, Ankara, Turkey; 2 Faculty of Health Sciences, Department of Nutrition and Dietetics, Ondokuz Mayis University, Samsun, Turkey

**Keywords:** COVID-19, Nutrition, Fear, Anxiety, Nutrition habits

## Abstract

**Objective:**

The aim of this study was to evaluate effects of fear and anxiety on nutrition during the COVID-19 pandemic.

**Design:**

Participants were recruited by an online survey in this cross-sectional study. The questionnaire included general demographic characteristics, level of fear and anxiety, and nutritional habits. The Fear of COVID-19 Scale (FCV-19S) and Generalized Anxiety Disorder-7 test (GAD-7) were used to determine fear and anxiety.

**Setting:**

Turkey.

**Participants:**

A total sample consisted of 1012 adults.

**Results:**

In pandemic, fear and anxiety caused individuals to skip breakfast and snacks less, but more at lunch. A positive significant correlation was observed between the increased consumption of yoghurt, cheese and water and FCV-19S scores. There was a positive significant correlation between cheese, legume, nuts-seeds, cake-cookies, dessert and tea consumption and GAD-7 scores. A 1-unit increase in FCV-19S scores affected 1·04 times of increased consumption of yoghurt, kefir, cheese, nuts-seeds, fruit (dry) and rice-pasta. A 1-unit increase in GAD-7 scores affected 1·03 times of increased consumption of egg and fruit (fresh); 1·04 times of increased consumption of cheese and other vegetables; 1·05 times of increased consumption of milk, meat, poultry, fish, legume, nuts-seeds, fruit (dry), cake-cookies and tea; 1·07 times of increased consumption of rice-pasta and coffee and 1·08 times of increased consumption of bread and dessert.

**Conclusions:**

In pandemic, anxiety and fear led to changes in individuals’ nutritional habits and food preferences. Continuous surveillance of psychological consequences for outbreaks should become routine as part of preparedness efforts worldwide. In addition, the effects of these psychological problems on nutrition should be evaluated.

## Graphical abstract

**Figure:**
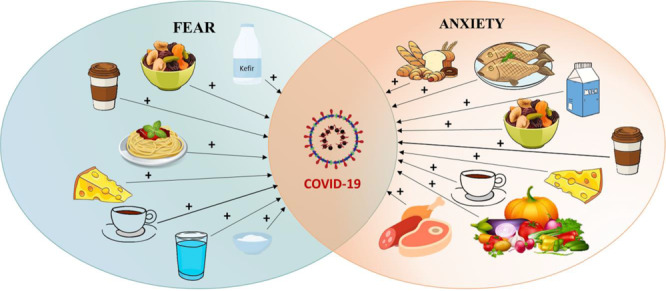

The coronovirus pandemic occurred as a pneumonia pandemic in Wuhan, Hubei Province, China in December 2019 and was later named the COVID-19 by the WHO^(1)^. The pandemic is transmitted from animal to person and progress with severe acute respiratory problems^(2)^. Despite its occurrence in China, the COVID-19 pandemic, which tends to spread rapidly in the world, has become a major public health problem in many countries and has been accepted as a global health problem that requires urgent action^(3)^. As of July 16, the number of confirmed cases worldwide has exceeded 13 million^(4)^. In Turkey, on the 16 July 2020, 216·873 cases has been identified and 5·440 patients died from the COVID-19^(5)^.

The current treatment is mainly focused on infection control and effective vaccine therapy because of extensive measures to reduce person-to-person transmission of the COVID-19^(6,7)^. But the COVID-19 pandemic poses serious threats to individuals’ physical health and life. The psychosocial repercussions of the COVID-19 pandemic have not yet been fully considered. The COVID-19 pandemic also triggered a wide range of psychological problems, including panic attacks, anxiety, fear, depression, stress and insomnia^(8–10)^. In addition to the COVID-19 pandemic, it has implications for other spheres: family organisation, closings of schools/universities, companies and public places, changes in work routines, isolation, the implementation of the law and limits of quarantina, leading to feelings of helplessness and abandonment^(11)^. When evaluated from this point of view, infectious diseases cause fear as well as depression and anxiety^(12)^.

Depending on the psychological problems, there may also be changes in nutritional status and habits. The stress period affects the amount and type of food that most people eat. In various studies conducted on adults, approximately 35–60 % of people ate more total energies during stress period, while 25–40 % of people ate less^(13,14)^. It is thought that there may be differences in dietary habits due to people having difficulty in food supply during quarantine, spending more time at home and anxiety and fear developing due to the COVID-19 pandemic. It is an important factor to consider the effect of lifestyle change, including unhealthy food preferences and dietary habits, on susceptibility and recovery to the COVID-19 infection. As far as we know, there is no research examining the effect of the psychological effect caused by the epidemic of the COVID-19 on nutritional habits. Therefore, in this study, nutritional attitudes and habits that changed due to fear and anxiety status were evaluated during the COVID-19 pandemic.

## Methods

### Study design and subjects

The study data were collected between 15 April and 30 April as an online survey. By utilising convenience sampling, we have reached 1012 participants in Turkey including in the cities where the pandemic is widespread. Adult individuals aged 18–65 years were included in the study. A total of twenty-eight questionnaire data were excluded from the study consisting of individuals under 18 and over 65 years old and incomplete survey.

### Ethical approval

This study, in which participants participated on a voluntary basis, was conducted in accordance with all ethical procedures/standards and the Declaration of Helsinki. The study was approved by the Turkey’s Health Ministry (Approval number: 2020-05-04T21_13_39) and Ondokuz Mayıs University Clinical Research Ethics Committee (Approval number: B.30.2.ODM.0.20.08/257).

### Research instruments

In this study, including demographic information of the participants (such as gender, age, height, weight, BMI, educational status, marital status, working status, chronic illness and smoking), the Fear of COVID-19 Scale (FCV-19S), Generalized Anxiety Disorder-7 test (GAD-7) and questionnaire which prepared by researchers contain change nutritional attitudes and habits (such as the number of meals, snacks, changes in food preferences and supplements) in this period were administered.

### The fear of COVID-19 Scale

The FCV-19S is a unidimensional seven-item, five-point Likert scale, developed by Ahorsu *et al.* ^(15)^. First, a Turkish validity and reliability study was conducted, and the FCV-19S was analysed with item total correlations (>0·5) and Cronbach alpha internal consistency coefficients (0·874)^(16)^. The higher the score, the greater the fear of COVID-19. In this study, the reliability coefficient of the scale was found *α* = 0·87.

### The Generalized Anxiety Disorder-7 test

The GAD-7 test is a unidimensional seven-item, four-point Likert scale, developed by Spitzer *et al.* ^(17)^. Score between 0 and 4 was mild, 5–9 was moderate, 10–14 was high and 15–21 was considered as serious anxiety. For the GAD-7 test total score, eight cut-off values were determined. The Turkish version of the GAD-7 test has previously been shown to have high sensitivity and validity^(18)^. In this study, the reliability coefficient of the scale was found *α* = 0·89.

### Evaluation of nutritional attitudes and habits

During the COVID-19 pandemic period prepared by the researchers, a thirty-two-item questionnaire was created that questions food selection and habits. During the COVID-19 epidemic period, participants were questioned about pretreatments in food preparation, different cleaning procedures after buying food, skipping meals and food preferences (dairy products, meat and meat products, vegetables and fruits, grains, beverages and nutrition supplements).

### Statistical analysis

Histogram, q-q plots and Shapiro–Wilk’s test are examined to assess the data normality. Levene test is used to test variance homogeneity. Kruskal–Wallis *H* test is applied to compare the distribution of FCV-19S and GAD-7 test scores among food consumption categories. Bonferroni-adjusted Dunn test is used for *post hoc* analysis. To examine the relationship between food consumption and FCV-19S and GAD-7 test scores, Kendall tau-b correlation coefficients are also calculated. The relationship between outdoor market-grocery-online shopping, cleaning foods, cleaning vegetable and fruits and FCV-19S and GAD-7 test scores is evaluated using Mann–Whitney *U* test and point biserial correlation coefficient. The coefficients are interpreted as follows: 0–0·30 very weak correlation, 0·31–0·50 weak correlation, 0·51–0·70 moderate correlation, 0·71–0·90 high correlation and 0·91–1·00 very high correlation. Since proportional odds assumption of ordinal logistic regression analysis is not met, binary logistic regression analysis models are built separately for increased and decreased food consumption to identify the influence of FCV-19S and GAD-7 test scores on the outcomes. Both crude and adjusted models are fitted. Adjusted models are built by controlling the effect of age, gender, smoking status, current working status, education status, marital status and BMI. To control for multiple testing, all *P* values are adjusted using Benjamini–Hochberg procedure. Adjusted *P* values <5 % are considered as statistically significant. All analyses are conducted using R 3.5.1^(19)^ and TURCOSA (Turcosa Analytics Ltd Co.^(20)^) software.

## Results

In this study, effects of anxiety and fear on nutrition were evaluated during the COVID-19 pandemic among 1012 individuals (*n* 827 female, *n* 185 male) living in Turkey. Basic demographic characteristics of study participants are shown in Table 1. An average of age was 28·3 ± 8·7 years old, and 87·6 % of whom have an undergraduate degree education level. Average BMI, the FCV-19S scores and the GAD-7 test scores were 23·5 ± 4·7 kg/m^2^, 19·1 ± 6·3 and 5·7 ± 4·8, respectively. In this period, 26·7 % of individuals use nutritional supplements and the most used food supplement was multivitamin-mineral (9·0 %).

**Table 1:** Basic demographic characteristics of study participants

Characteristics	Statistics	
	*n*	%
Age (years)		
Mean	28·3	
sd	8·7	
Gender (female)	827	81·7
Marital status (married)	337	33·3
Education level		
Literate	7	0·7
Primary school	30	3·0
High school	88	8·7
Undergraduate or graduate	887	87·6
Smoking (yes)	153	15·1
Current working status		
Not working	525	51·9
Unpaid vacation	65	6·4
Paid vacation	126	12·5
Flexible overtime	158	15·6
Working	129	12·7
Dismissal	9	0·9
Chronic disease (available)	104	10·3
Nutritional supplements (available)	270	26·7
Multivitamin-mineral	91	9·0
Vitamin C	41	4·1
Vitamin D	25	2·5
Vitamin B_12_	4	0·4
Zn	19	1·9
*n*-3	14	1·4
Krill oil	1	0·1
Probiotic	7	0·7
Turmeric extract	1	0·1
Nigella sativa	9	0·9
*β*-Glucan	6	0·6
Propolis	25	2·5
Black elderberry	10	1·0
Reishi mushroom	1	0·1
BMI (kg/m^2^)		
Mean	23·5	
sd	4·7	
FCV-19S scores		
Mean	19·1	
sd	6·3	
GAD-7 test scores		
Mean	5·7	
sd	4·8	

BMI: Body Mass Index; GAD, Generalized Anxiety Disorder; FCV-19S, Fear of COVID-19 Scale.

Distribution of individuals according to their skipping status is given in Table 2. While individuals skipped less breakfast and snacks during the COVID-19 pandemic, the frequency of skipping lunch increased significantly (*P* < 0·05).

**Table 2:** Meal skips before and during the pandemic

Meals	Before		During		Chi-Square	*P*
	*n*	%	*n*	%		
Breakfast	201	19·9	169	16·7	4·903	0·027^*^
Lunch	360	35·6	498	49·2	56·533	<0·001^*^
Dinner	31	3·1	33	3·3	0·025	0·874
Snacks	555	54·8	360	35·6	131·136	<0·001^*^

* *P* < 0·01, McNemar test.

**Fig. 1:**
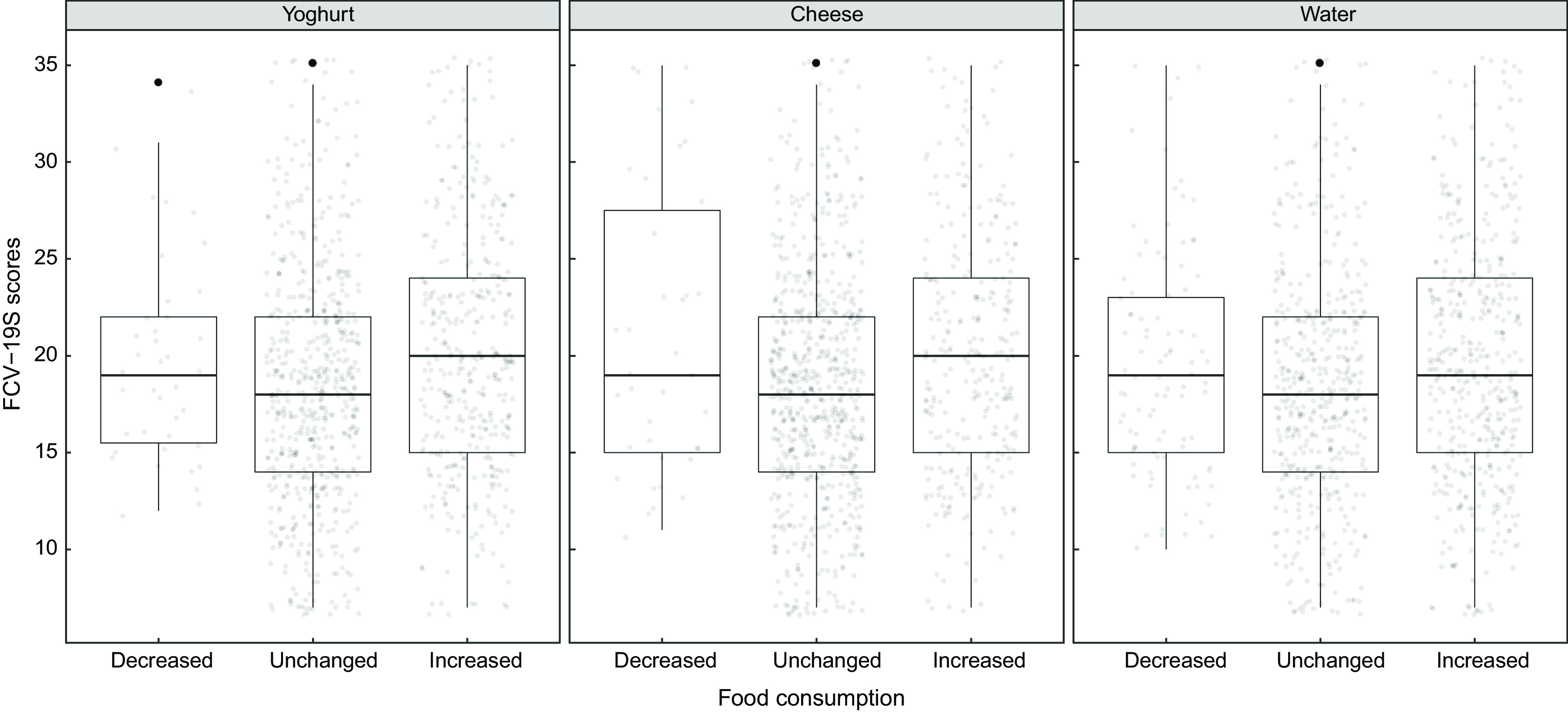
The relationship between food consumption and Fear of COVID-19 Scale (FCV-19S)

Investigation of the relationship between food consumption and FCV-19S and GAD-7 test scores is shown in Table 3. A significant difference was found in the distribution of individuals’ FCV-19S scores for yoghurt, cheese and water consumption (*adj.P* < 0·05). It was observed that yoghurt, cheese and water consumption increased in individuals with high FCV-19S scores distribution. A positive, weak, significant correlation was observed between the increased consumption of yoghurt, cheese and water and the FCV-19S scores (respectively, tau-b = 0·087, 0·064 and 0·053, *adj.P* < 0·05) (Fig. 1). A significant difference was found in the distribution of individuals’ FCV-19S scores for consumption of kefir, nuts-seeds, fruit (dry), rice-pasta and coffee (*adj.P* < 0·05). In individuals with high distribution of FCV-19S scores, consumption of kefir increased; nuts-seeds, rice-pasta and coffee consumption decreased. However, there was no significant correlation between these foods and the FCV-19S scores (*adj.P* > 0·05) (Table 3).

**Table 3:** Investigation of the relationship between food consumption and Fear of COVID-19 Scale (FCV-19S) and Generalized Anxiety Disorder-7 test (GAD-7) test scores

Foods	FCV-19S scores								GAD-7 test scores							
	Food consumption						*adj.p*	Kendall tau-b	Food consumption						*adj.p*	Kendall tau-b
	Decreased		Unchanged		Increased				Decreased		Unchanged		Increased			
	Median	1st–3rd quartiles	Median	1st–3rd quartiles	Median	1st–3rd quartiles			Median	1st–3rd quartiles	Median	1st–3rd quartiles	Median	1st–3rd quartiles		
Dairy products																
Milk	19	17–23	19	15–22	19	14–24	0.158	0.011	7	3–10^**a**^	4	2–7^**b**^	5	3–9^**a**^	**0.001**	0.024
Yoghurt	19	15–22^**ab**^	18	14–22^**a**^	20	15–24^**b**^	**0.011**	0.087**	7	4–11^**a**^	4	2–7^**b**^	5	2–8^**b**^	**0.001**	0.009
Kefir	20	18–25^**ab**^	18	14–23^**a**^	20	16–24^**b**^	**0.025**	0.011	6	3–12^**a**^	4	2–8^**b**^	5	3–9^**ab**^	**0.019**	–0.015
Cheese	19	15–28^**ab**^	18	14–22^**a**^	20	15–24^**b**^	**0.014**	0.064*	7	3–11^**a**^	4	2–7^**b**^	5	3–9^**a**^	**0.001**	0.051*
Meat and meat products																
Meat	19	14–25	18	15–22	20	15–23	0.368	0.012	6	3–10^**a**^	4	2–7^**b**^	6	3–9^**a**^	**0.001**	0.025
Poultry	19	16–23	18	15–23	19	14–24	0.262	–0.005	6	3–10^**a**^	4	2–7^**b**^	6	3–9^**a**^	**0.001**	0.005
Fish	19	15–24	19	15–23	19	15–24	0.277	–0.008	5	3–10^**a**^	4	2–7^**b**^	5	3–9^**a**^	**0.006**	–0.005
Egg	20	16–27	18	14–22	19	15–24	0.143	0.014	7	5–11^**a**^	4	2–7^**b**^	5	2–8^**c**^	**0.001**	0.018
Legume	20	15–27	18	15–22	19	15–24	0.246	0.026	6	4–10^**a**^	4	2–7^**b**^	5	3–9^**a**^	**0.001**	0.061*
Nuts-seeds	20	16–27^**a**^	18	14–22^**b**^	19	15–24^**ab**^	**0.036**	0.014	6	3–10^**a**^	4	2–7^**b**^	5	3–9^**a**^	**0.001**	0.055*
Vegetables and fruits																
Dark green vegetables	20	14–23	19	14–22	19	15–23	0.334	0.017	5	2–11	4	2–7	5	2–8	0.057	0.019
Other vegetables	20	15–24	18	15–22	19	15–23	0.277	0.013	6	4–11^**a**^	4	2–7^**b**^	5	2–9^**a**^	**0.001**	0.033
Fruit (fresh)	19	15–23	19	14–22	19	15–23	0.572	0.024	5	2–9	4	2–7	5	2–8	0.071	0.051
Fruit (dry)	20	16–27^**a**^	18	14–22^**b**^	20	16–24^**a**^	**0.016**	0.023	7	3–12^**a**^	4	2–7^**b**^	5	3–9^**a**^	**0.001**	0.031
Grain																
Bread	19	15–25	18	14–22	19	15–23	0.134	0.008	5	2–10^**a**^	4	2–7^**b**^	6	3–10^**a**^	**0.001**	0.047
Rice-pasta	20	15–26^**a**^	18	14–22^**b**^	20	16–24^**ab**^	**0.017**	–0.003	5	2–10^**a**^	4	2–7^**b**^	6	3–10^**a**^	**0.001**	0.035
Cake-cookies	19	14–24	18	14–22	19	15–23	0.339	0.012	4	2–8^**ab**^	4	1–7^**a**^	5	3–9^**b**^	**0.001**	0.083**
Dessert	19	15–24	18	14–22	19	15–24	0.246	0.028	4	1–8^**a**^	4	2–7^**a**^	6	3–10^**b**^	**0.001**	0.119**
Beverages																
Tea	21	16–23	18	14–22	19	15–23	0.068	0.020	5	2–7^**ab**^	4	2–7^**a**^	5	3–9^**b**^	**0.001**	0.088**
Coffee	20	15–25^**a**^	18	14–22^**b**^	19	15–24^**ab**^	**0.011**	–0.008	6	2–10^**a**^	4	2–7^**b**^	5	3–10^**a**^	**0.001**	0.048
Water	19	15–23^**ab**^	18	14–22^**a**^	19	15–24^**b**^	**0.037**	0.053*	6	4–11^**a**^	4	2–7^**b**^	4	2–8^**b**^	**0.001**	–0.023

Different superscripts in the same row indicate a statistically significant difference between groups.

Kendall tau-b coefficient is significant at adj.* *P* < 0.05, adj.** *P* < 0.01, adj.***P < 0.001.

All significant adjusted *P* values are shown in bold.

**Fig. 2:**
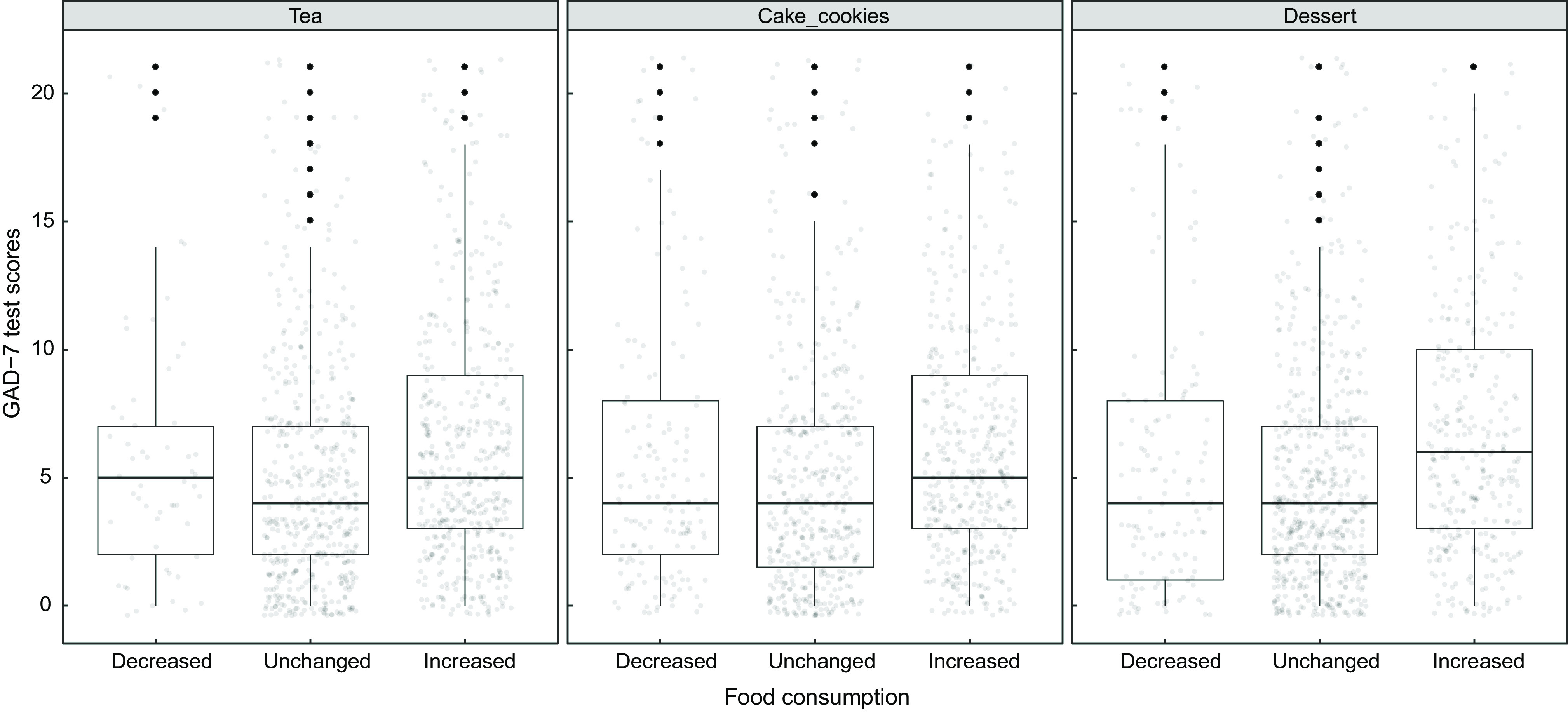
The relationship between food consumption and Generalized Anxiety Disorder-7 test (GAD-7) test scores

A significant difference was found in the distribution of individuals’ GAD-7 test scores in terms of cheese, legume, nuts-seeds, cake-cookies, dessert and tea consumption (*adj.P* < 0·05). Individuals with high GAD-7 test scores changed their consumption of cheese, legume and nuts-seeds, and consumption of cake-cookies, dessert and tea was increased. There was a positive, weak, significant correlation between cheese, legume, nuts-seeds, cake-cookies, dessert and tea consumption and the GAD-7 test scores (respectively, tau-b = 0·051, 0·061, 0·055, 0·083, 0·119 and 0·088, *adj.P* < 0·05) (Fig. 2). In the distribution of individuals’ GAD-7 test scores for other dairy products (milk, yoghurt and kefir), meat products (meat, poultry, fish and egg), other vegetables, fruit (dry), bread, rice-pasta, coffee and water consumption, there was a significant difference (*adj.P* < 0·05). However, no significant correlation was found between these foods and the GAD-7 test scores (*adj.P* > 0·05) (Table 3).

Investigation of the relationship between food shopping behaviour and FCV-19S and GAD-7 test scores is shown in Table 4. A significant difference was found in the distribution of individuals’ FCV-19S scores for making the food shopping from the outdoor market (*adj.P* < 0·05). The distribution of FCV-19S scores is high for those who do not shop for the food shopping from outdoor market. There was a negative, very weak, significant correlation between the outdoor market shopping and the FCV-19S scores (tau-b = −0·079 *adj.P* < 0·05). There was a significant difference between the distribution of individuals’ FCV-19S scores with disinfecting the food after buying and bringing it home, and especially vegetables and fruits cleaning application (such as vinegar water) (*adj.P* < 0·05). The FCV-19S scores were high in those who disinfect the food after buying it and bring it home and cleaning the vegetables and fruits (such as vinegar water). A positive, very weak, significant correlation was observed between the FCV-19S scores with the disinfection of the food after buying and bringing it home and cleaning the vegetables and fruits (respectively, tau-b = 0·077 and 0·117, *adj.P* < 0·05). In the COVID-19 pandemic, no significant difference was found in the distribution of GAD-7 test scores in terms of the places where people food shopping and the cleaning procedures they apply to the foods (*adj.P* > 0·05).

**Table 4:** Investigation of the relationship between food shopping behaviour and Fear of COVID-19 Scale (FCV-19S) and Generalized Anxiety Disorder-7 test (GAD-7) test scores

Variables	FCV-19S scores		GAD-7 test scores	
	Median	1st–3rd quartiles	Median	1st–3rd quartiles
Behaviour				
Outdoor market shopping				
No (*n* 725)	19	15–23	5	2–8
Yes (*n* 287)	17	14–22	4	2–8
*adj.P*	0·005		0·615	
*r*_*pb*_	–0·079*		–0·016	
Grocery shopping				
No (*n* 74)	20	15–23	5	2–7
Yes (*n* 938)	19	15–23	4	2–8
*adj.P*	**0·463**		0·885	
*r*_*pb*_	**–0·025**		0·003	
Online shopping				
No (*n* 802)	19	15–23	4	2–8
Yes (*n* 210)	18	14–23	5	2–8
*adj.P*	0·234		0·688	
*r*_*pb*_	–0·041		0·022	
Cleaning foods				
No (*n* 164)	18	14–22	5	2–8
Yes (*n* 848)	19	15–23	4	2–8
*adj.P*	**0·047**		0·979	
*r*_*pb*_	0·077*		0·002	
Cleaning vegetables and fruits				
No (*n* 216)	18	14–21	5	2–8
Yes (*n* 796)	19	15–23	4	2–8
*adj.P*	**0·005**		0·678	
*r*_*pb*_	0·117**		0·021	

Point biserial correlation coefficient is significant at *adj.* *P < 0·05, *adj.* **P < 0·01.

All significant adjusted *P* values are shown in bold.

Binary logistic regression results in identifying the influence of FCV-19S and GAD-7 test scores on increased food consumption are shown in Table 5. A 1-unit increase in the FCV-19S scores affected 1·04 times of increased consumption of yoghurt, kefir, cheese, nuts-seeds and fruit (dry) either before or after controlling for potential confounders (age, gender, current working status, education level, marital status and BMI) (*adj.P* < 0·05). A 1-unit increase in the FCV-19S scores affected 1·03 times of increased consumption of rice-pasta (*adj.P* < 0·05). When the effect of age, gender, current working status, education level, marital status and BMI variables is adjusted, this effect is 1·04 times and significant (*adj.P* < 0·05). A 1-unit increase in the FCV-19S scores affected 1·02 times of increased consumption of tea, and 1·03 times of increased consumption of coffee and water (*adj.P* < 0·05).

**Table 5:** Binary logistic regression results in identifying the influence of Fear of COVID-19 Scale (FCV-19S) and Generalized Anxiety Disorder-7 test (GAD-7) test scores on increased food consumption

Foods	FCV-19S scores						GAD-7 test scores					
	Crude			Adjusted^*^			Crude			Adjusted^*^		
	OR	95 % CI	*adj.P*	OR	95 % CI	*adj.P*	OR	95 % CI	*adj.P*	OR	95 % CI	*adj.P*
Dairy products												
Milk	1·02	0·99, 1·05	0·114	1·02	0·99, 1·05	0·164	1·05	1·02, 1·08	**0·004**	1·04	1·01, 1·08	**0·025**
Yoghurt	1·04	1·02, 1·07	**0·011**	1·04	1·02, 1·06	**0·011**	1·02	0·99, 1·05	0·123	1·02	0·99, 1·05	0·159
Kefir	1·04	1·01, 1·07	**0·049**	1·04	0·99, 1·08	0·079	1·04	0·99, 1·08	0·078	1·05	1·00, 1·09	**0·045**
Cheese	1·04	1·02, 1·06	**0·011**	1·04	1·02, 1·07	**0·011**	1·04	1·01, 1·07	**0·008**	1·03	1·00, 1·06	**0·044**
Meat and meat products												
Meat	1·02	0·99, 1·04	0·177	1·02	0·99, 1·05	0·172	1·05	1·02, 1·08	**0·004**	1·05	1·01, 1·08	**0·016**
Poultry	1·02	0·99, 1·05	0·149	1·02	0·99, 1·05	0·164	1·05	1·02, 1·08	**0·005**	1·04	1·00, 1·07	**0·049**
Fish	1·03	0·99, 1·06	0·114	1·03	0·99, 1·06	0·164	1·05	1·01, 1·09	**0·014**	1·05	1·01, 1·10	**0·021**
Egg	1·02	0·99, 1·04	0·114	1·01	0·99, 1·04	0·259	1·03	1·00, 1·06	**0·035**	1·02	0·99, 1·05	0·148
Legume	1·02	0·99, 1·04	0·114	1·02	0·99, 1·04	0·164	1·05	1·02, 1·08	**0·002**	1·04	1·01, 1·07	**0·013**
Nuts-seeds	1·04	1·02, 1·07	**0·011**	1·04	1·01, 1·07	**0·016**	1·05	1·02, 1·09	**0·002**	1·05	1·01, 1·08	**0·015**
Vegetables and fruits												
Dark green vegetables	1·02	0·99, 1·04	0·114	1·01	0·99, 1·03	0·399	1·02	0·99, 1·05	0·184	1·01	0·98, 1·04	0·710
Other vegetables	1·02	0·99, 1·04	0·114	1·02	0·99, 1·04	0·236	1·04	1·01, 1·06	**0·020**	1·02	0·99, 1·05	0·136
Fruit (fresh)	1·01	0·99, 1·04	0·149	1·01	0·99, 1·03	0·281	1·03	1·01, 1·06	**0·022**	1·02	0·99, 1·05	0·136
Fruit (dry)	1·04	1·02, 1·07	**0·011**	1·04	1·01, 1·07	**0·016**	1·05	1·02, 1·09	**0·002**	1·05	1·01, 1·08	**0·015**
Grain												
Bread	1·02	0·99, 1·05	0·103	1·02	0·99, 1·05	0·164	1·08	1·05, 1·11	**0·002**	1·06	1·03, 1·10	**0·007**
Rice-pasta	1·03	1·01, 1·06	**0·046**	1·04	1·01, 1·06	**0·042**	1·07	1·03, 1·10	**0·002**	1·05	1·02, 1·09	**0·013**
Cake-cookies	1·02	0·99, 1·04	0·177	1·01	0·98, 1·03	0·566	1·05	1·02, 1·08	**0·002**	1·04	1·01, 1·07	**0·033**
Dessert	1·02	0·99, 1·05	0·079	1·01	0·99, 1·04	0·173	1·08	1·05, 1·11	**0·002**	1·06	1·03, 1·10	**0·007**
Beverages												
Tea	1·02	1·00, 1·04	**0·049**	1·02	0·99, 1·04	0·164	1·05	1·02, 1·08	**0·002**	1·04	1·01, 1·07	**0·016**
Coffee	1·03	1·00, 1·05	**0·048**	1·03	1·00, 1·05	0·075	1·07	1·04, 1·11	**0·002**	1·06	1·03, 1·09	**0·007**
Water	1·03	1·01, 1·05	**0·017**	1·03	1·00, 1·05	0·067	1·02	0·99, 1·05	0·237	1·00	0·98, 1·03	0·840

All significant adjusted *P* values are shown in bold.

* Adjusted by age, gender, smoking status, current working status, education status, marital status and BMI.

A 1-unit increase in the GAD-7 test score affected 1·03 times of increased consumption of egg and fruit (fresh); 1·04 times of increased consumption of kefir, cheese and other vegetables; 1·05 times of increased consumption of milk, meat, poultry, fish, legume, nuts-seeds, fruit (dry), cake-cookies and tea; 1·07 times of increased consumption of rice-pasta and coffee and 1·08 times of increased consumption of bread and dessert (*adj.P* < 0·05). As the GAD-7 test scores of individuals increased, a significant difference was found between the increase in consumption of milk, kefir, cheese, meat, poultry, fish, legume, nuts-seeds, fruit (dry), bread, rice-pasta, cake-cookies, dessert, tea and coffee either before or after controlling for potential confounders (age, gender, current working status, education level, marital status and BMI) (*adj.P* < 0·05).

## Discussion

This study was planned to investigate individuals’ attitudes and habits of nutrition during the COVID-19 pandemic in Turkey. This association remained significant even after controlling for a wide range covariates. To the best of our knowledge, this is the first study during the COVID-19 pandemic to examine the association between anxiety, fear and nutritional habits.

Nutritional supplements can be used to improve individuals’ results and regulate the inflammatory response during lung infection. Among these, antioxidants play an important role in protecting lung cells against bacteria and viruses. Several studies reported the protective role of the antioxidants in lung infection and lung inflammation^(21–23)^. In our study, it was determined that 26·7 % of the participants used in particular vitamin C, vitamin D, vitamin B_12_ and Zn (Table 1). It may be because the participants believed that the use of nutritional supplements in about a quarter of the COVID-19 epidemic, which is pneumonia, had a protective role in lung infections, being immune modulators and inflammatory mediators.

During the COVID-19 pandemic, due to the limited or strict quarantine process implemented in our country, people had to spend most of their time at home. It has been determined that the participants had more regular breakfast and snacks during the COVID-19 epidemic, which they skipped due to the pre-pandemic working status, time limitation, etc. However, it is thought that the participants skipped lunch due to changes in sleep time and having breakfast late (Table 2). SARS-CoV-2 can survive from 3 h to 72 h on various surfaces such as plastic, stainless steel, copper, cardboard and aerosols^(24)^. In our study, it has been determined that there are differences in shopping preferences, processes applied to foods and vegetables and fruits due to the fear of virus contamination from the surfaces (Table 4).

The mechanisms between psychological problems and dietary intake are unknown. Psychological problems, in particular depression, fear and anxiety, are the result of interaction between genetic, hormonal, immunological, biochemical and neurodegenerative factors^(25)^. A study in Iranian adults found an inverse relationship between anxiety and greater consumption of vegetables and fruits, legume and dairy products^(26)^. The ATTICA work (was a health and nutrition survey conducted in the province of Attica, Greece) focused only in women reported that increased consumption of sweets and meat products was associated with higher anxiety levels^(27)^. Another study reported that there was an inverse relationship between high intake of vegetables, fruits and anxiety^(28)^. In our study, there was a significant difference between the individuals’ FCV-19S scores and yoghurt, cheese and water consumption, whereas the GAD-7 test scores and cheese, legume, nuts-seeds, cake-cookies, dessert and tea consumption were found to be significant. In addition, as the FCV-19S scores increase, a significant difference was found between the increase in consumption of yoghurt, kefir, cheese, nuts-seeds, fruit (dry), cake-cookies, tea coffee and water consumption, the GAD-7 test scores increase, a significant difference was found between the increase in consumption of milk, kefir, cheese, meat, poultry, fish, legume, nuts-seeds, fruit (dry), bread, rice-pasta, cake-cookies, dessert, tea and coffee. The associations are partly in line with the literature, where mixed results are reported.

One of the strengths of this study was its large population. We made adjustment for several potential confounders, and identified associations were independent of these factors. However, some limitations deserve mentioning. First, the nature of our study was cross-sectional, and therefore, we cannot provide a causal link between pandemic-related fear and anxiety and nutritional habits. Second, the findings of this study were based on self-report data which has the risk of source bias.

## Conclusion

In conclusion, we found evidence indicating an inverse association between anxiety, fear and nutrition habits during the COVID-19 pandemic. Continuous surveillance of the psychological consequences for outbreaks should become routine as part of preparedness efforts worldwide. In addition, the effects of these psychological problems on nutrition should be evaluated. Further studies, in particular with prospective design, are required to confirm our findings.
